# Awareness and Sources of Knowledge about Obstructive Sleep Apnea: A Cross Sectional Survey Study

**DOI:** 10.3390/healthcare11233052

**Published:** 2023-11-27

**Authors:** Maria Lavinia Bartolucci, Serena Incerti Parenti, Francesco Bortolotti, Tommaso Gorini, Giulio Alessandri-Bonetti

**Affiliations:** Unit of Orthodontics and Sleep Dentistry, Department of Biomedical and Neuromotor Sciences (DIBINEM), University of Bologna, Via San Vitale 59, 40125 Bologna, Italy; maria.bartolucci3@unibo.it (M.L.B.); francesco.botolott4@unibo.it (F.B.); tommi.gorini@gmail.com (T.G.); giulio.alessandri@unibo.it (G.A.-B.)

**Keywords:** obstructive sleep apnea, source of knowledge, sleep disordered breathing, level of knowledge

## Abstract

Obstructive sleep apnea (OSA) is a multifactorial sleep breathing disorder, seriously impacting quality of life and involving approximately 1 billion of the world’s population. It is characterized by episodes of total cessation of breathing or decreases in airflow during sleep. Available data suggest that most cases of OSA remain undiagnosed even in developed countries. This is due to a lack of widespread knowledge about this pathology and the medical morbidities and mortality it brings about, among both laypeople and physicians. Moreover, despite receiving indications about the need to undergo specific evaluations for OSA signs and symptoms, sometimes patients do not pay sufficient attention to the problem. This is probably due to a lack of correct information on these issues. The present investigation analyzed the level of knowledge about OSA pathology and the sources through which a group of OSA patients gained information on their condition. A survey of 92 patients diagnosed with OSA (mean age 60.55 ± 10.10) and referred to the Unit of Orthodontics and Dental Sleep Medicine of the University of Bologna was conducted by means of a questionnaire investigating sociodemographic characteristics, the level of general knowledge on OSA pathology and its possible medical consequences. Despite about two third (67.38%) of the population demonstrating extensive knowledge, remarkably, a group of subjects (20.65%) had poor awareness of the OSA condition. A statistically significant correlation emerged between the level of knowledge about OSA and the level of education (*p* = 0.002). A great effort should be made to improve the quality of information and the communication modalities for OSA to enable a fully appropriate awareness of the condition among patients.

## 1. Introduction

Approximately 1 billon of the world’s population has recently been estimated to be affected by obstructive sleep apnea (OSA), the most common type of sleep disordered breathing [1] characterized by repetitive partial (hypopnea) or complete (apnea) obstruction of the upper airways, lasting 10 s or more while sleeping, that entail continued or increased respiratory effort [2]. This condition causes periodic oxygen desaturation and recurrent arousal which leads to a fragmented and therefore poor sleep [3].

The pathogenesis of OSA is multifactorial and therefore it is not merely caused by obstructions of the upper airways. OSA is indeed also associated with functional abnormalities [4,5,6] such as inadequate upper airway muscle function that causes collapsibility of the pharynx, a large ventilatory response to a respiratory disorder (high loop gain) and a low arousal threshold which is the level of inspiratory effort at which obstructive events terminate with an arousal from sleep [7]. The respiratory system is supported by a complex interaction between the central nervous system, respiratory-related motor neurons, and the muscles of respiration. Both volitional and metabolic pathways are active during wakefulness in order to determine the minute ventilation necessary to maintain eucapnia (the CO_2_ level during stable breathing) [8]. At sleep onset, a great number of changes in the upper airway predispose the soft tissues to collapse: upper airway muscle activity decreases [9,10] with a consequent decrease in upper airway compliance, the lumen size of the airway becomes smaller and the upper airway resistance increases [11,12]. Moreover, this causes a reduction in the pharyngeal neurocompensatory reflexes (triggered by negative intraluminal pressure) and of ventilatory motor output (due to the loss of the wakefulness stimuli) [13]. This may result in sleep disordered breathing in predisposed individuals and the intermittent hypoxia entails an increased risk of morbidity and mortality for cardiovascular events, but also metabolic and neurocognitive impairments [14]. The prevalence of moderate to severe OSA (≥15 events per hour) in the general population aged ≥ 40 years is 23.4% in women and 49.7% in men, as observed in a large population-based sample of patients who underwent polysomnography. More specifically, after includ-ing excessive daytime sleepiness in the definition—with an apnoea–hypopnea index of five events per hour or more [15]—the prevalence of OSA was determined to be 12.5% in men and 5.9% in women, with an estimated one billion people between 30 and 69 years old being affected worldwide [1,16]. An accurate evaluation of the patient’s history is crucial for OSA identification. Snoring, gasping or choking and witnessed apneas are the most common complaints. In addition, excessive daytime sleepiness, impaired cognitive abilities, morning headaches, nocturia, and mood disorders are commonly reported by the patients. Undiagnosed OSA is a significant burden on the healthcare system, since untreated OSA patients more often need medical consultation, highlighting the importance of early and accurate diagnosis of this common disorder, to improve quality of life and also decrease motor vehicle accidents due to sleepiness [17].

Continuous positive airway pressure (CPAP) is considered the gold standard for the treatment of patients with moderate to severe OSA. It involves wearing a mask during sleep that is connected to a pump that delivers a constant airflow (pressure) to keep the airway open. Treatment with CPAP is highly effective at reducing the frequency of respiratory events, decrease daytime sleepiness, and improving quality of life [18]. A mandibular advancement device (MAD) is indicated for patients with mild to moderate OSA or primary snoring and also represents an accepted therapy for patients with severe OSA who do not respond to or are unable or unwilling to tolerate CPAP [19].

A recent investigation aimed at assessing the societal and economic burden of OSA in Italy, estimated that about 56% of the adult population (from 15 to 74 years of age) are affected by OSA but only 4% received a diagnosis, and only 2% were treated [1,20,21]. Since this condition is constantly rising along with the global increase in obesity, it requires appropriate consideration in prevention programs [16]. Instead, it is frequently undiagnosed because patients tend not to regard symptoms like snoring or excessive daytime sleepiness as a disorder and do not seek medical consultation. On the other hand, signs and symptoms of OSA are frequently underestimated by primary care physicians leading to potential undertreatment [22]. It is well established that a minimal education about sleep medicine is provided to physicians and dentists who cannot provide adequate sleep health guidance for their patients [23]. Recently, OSA knowledge gaps and competences have been evaluated, between a sample of dentists and a compilation of published physicians’ OSA knowledge scores [23]. Dental practitioners showed non-inferior knowledge on OSA compared to physicians. Since dentists have the possibility to evaluate their patients more often than other physician groups, they should be trained to suspect the presence of sleep disorders, and to recognize typical signs and symptoms that require further investigation. Nevertheless, a recent study concerning OSA screening in dental clinics showed that, despite receiving indications to consult a sleep medicine specialist for a suspicion of severe OSA, only 47.1% of the subjects underwent specific evaluations in the next 3 months [24]. This data contrasts with that which emerges from another paper concerning screening for arterial hypertension in dental clinics [25]. In fact, on the advice of the dentist, 97% of the subjects underwent cardiovascular examinations within 3 years; thus, suggesting an insensitivity to the OSA problem on the part of the population. A recent investigation performed on about 1300 subjects, reported that only 21% of the sample knew about the OSA condition but only 13% were capable of giving a definition of it [26]. The authors underlined the poor knowledge and awareness about OSA in the general population and encouraged a health education campaign to promote awareness of this serious condition. No data are available concerning the knowledge and awareness of OSA pathology among diagnosed patients. This represents another interesting point that would help to understand whether OSA patients appropriately receive information about their condition and the possible therapeutic approaches. Adherence to treatments is fundamental for clinical success and in this regard, providing comprehensive information about OSA physiopathology and consequences could increase patients’ compliance.

The present study aimed to analyze the level of knowledge about OSA pathology and the sources through which a group of OSA patients gained information on their condition. An evaluation of possible correlations between the patients’ level of knowledge about their OSA condition and the modality of information was also performed. 

## 2. Materials and Methods

### 2.1. Study Design

This cross-sectional study surveyed patients who consecutively presented at the Unit of Orthodontics and Sleep Dentistry, Department of Biomedical and Neuromotor Sciences (DIBINEM), University of Bologna, Italy, between April 2021 and April 2022 for the evaluation of treatment with MAD. The inclusion criteria were: aged over 18 years with an established diagnosis of OSA based on polygraphic study according to the criteria of the American Academy of Sleep Medicine (i.e., AHI ≥ 5 with symptoms/sequelae or AHI ≥ 15 regardless of associated symptoms) [27]. Subjects without a polygraphic diagnosis of OSA, subjects aged <18 years and non-self-sufficient individuals (necessitating material and psychological support due to physical problems for previous accidents) were excluded from the present study. The protocol was approved by the Ethical Committee CE-AVEC of the AUSL of Bologna (number of approval: 562-2021-OSS-AUSLBO), and written informed consent was signed by each participant. 

### 2.2. Survey Development

A questionnaire was created on the basis of a literature review performed on papers from January 2021 and December 2022. Two papers [28,29] presented questionnaires with different purposes than the present investigation but which were useful for the structure of the data collection sheet delivered to the patients. 

Researchers with experience in dental sleep medicine identified potentially important key themes for evaluation (i.e., definition and symptoms, health consequences and treatment options for OSA, as well as sources of knowledge about OSA). The first section of the questionnaire included six questions on sociodemographic characteristics such as age, sex, ethnicity, educational level, employment, and marital status. The second section was related to level of knowledge about OSA and possible responses included “yes”, “no”, and “do not know”. Subjects were asked whether snoring and apneas could be considered normal conditions and whether polysomnography could be considered a valid instrument for the diagnosis of OSA. The subsequent questions investigated health consequences of OSA, evaluating whether participants acknowledged diabetes, hypertension, stroke, and road accidents among them. Finally, subjects were asked whether there is any treatment to solve OSA and whether they knew CPAP was treatment option for OSA. A cutoff score of at least six correct answers was used to define a good knowledge about OSA and a score with less than or equal to four correct answers indicated poor knowledge. The questions of the second section of the questionnaire were the following:

1. Do you think snoring is a normal condition?
2. Are interruptions in breathing (apneas) during sleep, a normal condition?
3. Can polysomnography be a valid tool for the diagnosis of OSA?
4. Do you think that obstructive sleep apnea syndrome can lead to diabetes?
5. Do you think that obstructive sleep apnea syndrome can lead to arterial hypertension?
6. Do you think that obstructive sleep apnea syndrome can lead to myocardial infarction?
7. Can obstructive sleep apnea lead to an increased risk of road accidents?
8. To date, are there treatments that resolve the obstructive sleep apnea syndrome?
9. Have you ever heard of CPAP?

The third and final section was about the sources of information on OSA. The respondent was asked through which health professional and/or source he/she had become aware of OSA and which professional figure made him/her suspect that he/she was suffering from OSA. After verbal instructions were given, each individual was seated in a quiet area and was given 10 min to anonymously complete the questionnaires, which were marked by numeric codes. An investigator was available to explain the questions and to check the questionnaires for completeness.

### 2.3. Statistical Analysis

A descriptive statistical analysis was performed in order to evaluate the prevalence of the answers to the single questions of the questionnaire. A multinomial regression analysis was computed in order to evaluate whether the number of correct answers was related to sociodemographic variables and to the sources of knowledge about OSA. Considering as primary outcome the correlation between the level of knowledge and the main source of knowledge about OSA, the minimum requested sample size, calculated based on literature data [28], setting β error at 80% and α error at 5%, was 85 subjects.

## 3. Results

After the application of inclusion and exclusion criteria, 92 subjects were recruited for the present study (72.8% men and 27.2% women, mean age 60.55 ± 10.10). Table 1 reports the sample description and the sociodemographic variables.

**Figure 1:**
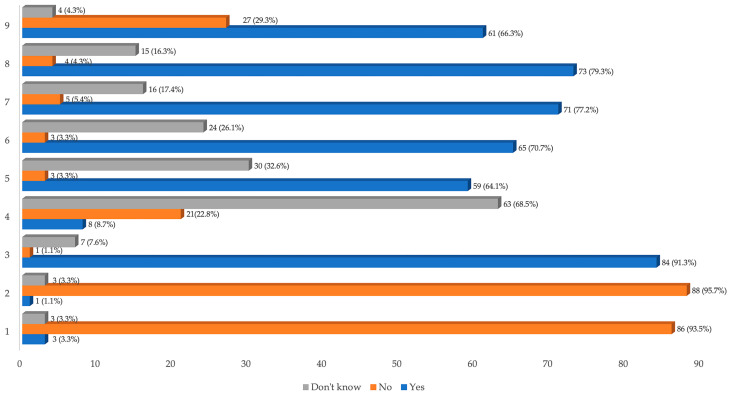
Results of the questionnaire on the level of knowledge about OSA. Notes: 1 = Do you think snoring is a normal condition? 2 = Are interruptions in breathing (apneas) during sleep a normal condition? 3 = Can polysomnography be a valid tool for the diagnosis of OSA? 4 = Do you think that obstructive sleep apnea syndrome can lead to diabetes? 5 = Do you think that obstructive sleep apnea syndrome can lead to arterial hypertension? 6 = Do you think that obstructive sleep apnea syndrome can lead to myocardial infarction? 7 = Can obstructive sleep apnea lead to an increased risk of road accidents? 8 = To date, are there treatments that resolve the obstructive sleep apnea syndrome? 9 = Have you ever heard of CPAP?

The educational level was quite high since 40.2% (n = 37) of the patients had a university degree and 41.3% (n = 38) had a high school diploma. As far as the level of knowledge on OSA pathology was concerned, about two thirds of the population (67.38%) had a score of six or higher in the questionnaire. On the other hand, about 20% of the study group had poor knowledge about OSA (four or less correct answers). Analyzing the answers to the single questions, most of the subjects (93.5%) showed a good general knowledge on the classification and diagnosis of OSA, while only 55.2% of the sample had received more specific information. Not all of the study group (72.8%) could properly report on the available treatment options for OSA. Only 66.3% of the patients reported being familiar with CPAP therapy, despite it currently representing the first line therapy for OSA. Table 2 reports the prevalence of the questionnaire score in the studied population and Figure 1 reports the results of the questionnaire about the level of knowledge about OSA.

**Figure 2:**
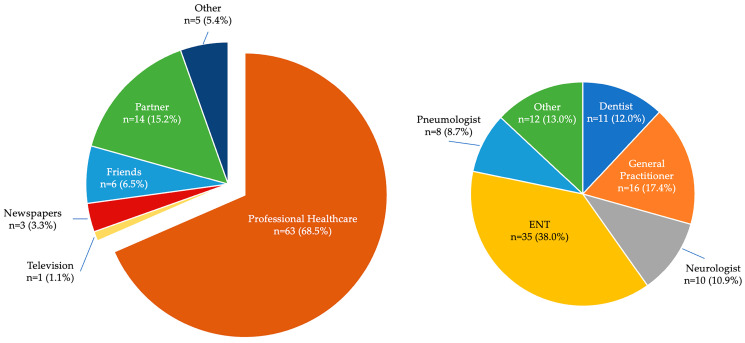
Sources of knowledge about OSA.

Regarding how the patients learned about OSA, Figure 2 shows that their first source of knowledge was a healthcare professional, followed by their bedroom partner, friends and newspapers. Among the healthcare professionals, the ENT is the one who presents and explains most about OSA to the patients (38%), followed by general practitioners and dentists. The first suspicion of the presence of OSA was suggested by the bed partner in most cases (46.7%). Being disturbed by the snoring, they usually search for information and then recommend specific investigations to the patients.

The multinomial regression analysis (Table 3) showed no correlation between the level of knowledge (number of correct answers) about the OSA condition and gender (*p* = 0.330), marital status (*p* = 0.677) and sources of knowledge (*p* = 0.852). A statistically significant correlation emerged between the level of knowledge about OSA and the level of education (*p* = 0.002).

## 4. Discussion

The present study investigated the sources and the level of knowledge about OSA pathology by means of a dedicated questionnaire, in a group of OSA patients referred to the Department of Orthodontics and Dental Sleep Medicine of the University of Bologna, in order to evaluate the possibility of performing MAD therapy. The mean age and the gender distribution of the sample were in line with the most recent epidemiological data on OSA pathology [15]. Considering the level of knowledge about OSA, a large number of subjects (67.38%) correctly answered the questionnaire, but approximately 20% of the sample was demonstrated to have a poor awareness of their condition having correctly answered a maximum of four questions (Table 2). This would mean that some patients who had been diagnosed with OSA, did not receive a clear explanation of their condition, or possible consequences and treatment options. In addition, the questions investigating more accurate information about OSA consequences such as cardiovascular or metabolic comorbidities, registered a higher number of wrong or uncertain answers, suggesting superficial knowledge in the general patient population. Promoting accurate information about OSA and related treatment options among people is crucial to facing this potentially life-threatening disease [26]. Sleep has a critical impact on health and wellbeing [30,31]. Its relevance has been emphasized during stressful situations such as the lockdown declared due to the COVID-19 pandemic when a deterioration in sleep patterns was observed [32].

The multinomial regression analysis did not show a significant correlation between the level of knowledge and the source of knowledge about OSA, but despite this, the role of physicians emerged as being essential. Indeed, most of the subjects were first informed about their condition by physicians and, moreover, all the patients were referred by physicians to the Department of Orthodontics and Dental Sleep Medicine for specialist assessment after receiving their diagnosis. Consequently, doctor–patient communication appears to have a central role in making the patient aware of his own pathology (Figure 2). This outcome is in agreement with the results of Senturk and coworkers [28] who concluded that health care professionals are the most reliable source of information for the OSA condition. General practitioners emerged as playing an important role in referring patients to sleep specialists for an accurate diagnosis (17.4%). This could be related to the more assiduous relationship these doctors have with their patients and to the numerous update courses that they follow. Among the specialists who most often identify the presence of OSA and adequately explain this condition to the patients, ENT (38.0%) and dentists (12.0%) experienced in sleep disorders emerged as having a relevant impact. Therefore, doctors who follow advanced educational and training programs appear to be more skilled in communicating with patients, making them aware of the pathophysiology of OSA, and also its consequences. Understanding possible comorbidities and adverse events brought about by OSA pathology is very important to achieving good compliance and adherence to treatment, which are essential conditions for therapeutic success. In this regard, a recent study concluded that patient education and personalized behavioral interventions may help increase CPAP adherence [33]. Similarly, MAD therapy also requires optimal compliance and informing patients about possible side effects is of crucial importance in order to avoid treatment interruptions [34]. Interesting data that emerges from the present investigation is represented by the significant correlation (*p* = 0.018) between the degree of patients’ awareness about OSA and their educational level. This represents an important issue to be addressed by clinicians while also explaining the condition: different patients require different language registers and attitudes.

The Internet is an important and easily accessible source of knowledge for health information: nowadays, this information-seeking strategy is considered an ideal supportive tool among laypeople willing to play an active role in healthcare decision-making. Interestingly, more than 80% of patients regard online resources as a powerful and influential tool for obtaining information about their health, with the potential to influence their treatment decisions and judgements [35,36]. Further potential is represented by audio-visual material that makes content and messages more engaging and much easier to remember for people compared with written information [37]. However, it is important to acknowledge that the health information provided by on-line videos is often scientifically inaccurate and misleading [38]. It has recently been demonstrated that the online written information provided by websites about OSA cannot be considered an adequate source of information for patients. The majority of websites fail to adequately address the most important aspects of MAD therapy, showing an overall suboptimal understandability for laypeople [39]. Likewise, the overall reliability, quality and completeness of the contents of online audio-visual information on the treatment of OSA with MAD are generally poor [40]. These data suggest that currently available online information might cause confusion among patients, negatively influencing their expectations and adherence to treatment. Therefore, experts in the field should endeavor to provide more accurate and complete contributions to online health information, while policymakers should consider developing a standardized peer-review process. More attention should be paid to reporting on the diagnosis, consequences and possible therapies for OSA in order to improve motivation and compliance [41]. Treatment duration, possible side effects and contraindications should be clearly addressed by means of quality content, in order to directly or indirectly convey the right message to possible OSA patients. Furthermore, mass media should be properly used to create a knowledge network involving people with different cultural levels among partners, friends and families. The importance of having a qualified physician and dentist involved in the multidisciplinary diagnostic and therapeutic process should also strongly emerge, not only to provide information to the patients but also to encourage medical practitioners to continuously update and train. All these aspects would aid patients in making timely treatment decisions, facilitating realistic expectations, and promoting good compliance. Considering the importance of OSA in terms of severity and diffusion, all the most popular media should be used to spread information across the population, overcoming cultural and age limits. Awareness-raising campaigns could also help to sensitize those who do not have the possibility of being advised by a doctor or alerted by a partner in order to reduce the percentage of people worrying who are estimated to be underdiagnosed [26]. The sample of the present study is not fully representative of the general OSA population, since the patients recruited were referred to the Orthodontics and Dental Sleep Medicine Section after they became eligible for MAD therapy. Another limitation of the study is that the survey did not investigate every possible specialist involved in OSA diagnosis and management, such as cardiologists and nutritionists. It would be interesting to evaluate this point in a future investigation and also extend the survey to a general OSA sample.

## 5. Conclusions

The results of the present study underline that the general knowledge of OSA pathology among a group of OSA patients was quite good. Nevertheless, 20% of the sample showed poor awareness of their condition. Furthermore, a lower knowledge emerged regarding more specific questions about the consequences of OSA and possible therapeutic options. A poor awareness was also found in subjects with a lower level of education. The first source of knowledge was represented by different physicians, in particular ENT specialists. Also, dentists who were experts in sleep medicine played a central role as diagnostic sentinel, underlining the importance of performing specific training programs for the management of OSA in patients. Considering the relevance of OSA pathology and the medical consequences that OSA can bring about, it is essential to improve the sources of knowledge available, promoting advanced educational and training programs among clinicians and aimed at increasing both the cultural level and communication skills. Also, more reliable and evidence-based media and web content should be provided to correctly inform laypeople, suggesting proper diagnostic workup and stressing the importance of undergoing specific evaluation with physicians and dentists experienced in sleep medicine.

## Figures and Tables

**Table 1:** Sample description.

**Gender**	Male	n = 67	72.8%
	Female	n = 25	27.2%
**Country of Origin**	Italy	n = 88	95.7%
	EU Country	n = 3	3.3%
	Not EU Country	n = 1	1.1%
**Level of Education**	Primary School	n = 1	1.1%
	Middle School Graduation	n = 16	17.4%
	High School Diploma	n = 38	41.3%
	University Degree	n = 37	40.2%
**Occupation**	Student	n = 0	0
	Government Employee	n = 20	21.7%
	Freelance	n = 15	16.3%
	Private Employee	n = 18	19.6%
	Unemployed	n = 0	0
	Retired	n = 33	35.9%
	Other	n = 6	6.5%
**Marital Status**	Single	n = 15	16.3%
	Divorced	n = 8	8.7%
	Married	n = 60	65.2%
	Engaged in a stable relationship	n = 9	9.8%

**Table 2:** Prevalence of the questionnaire score in the study population.

Score (Number of Correct Answers)	Prevalence %
9	0% (n = 0)
8	5.43% (n = 5)
7	34.78% (n = 32)
6	27.17% (n = 25)
5	11.96% (n = 11)
4	10.87% (n = 10)
3	7.61% (n = 7)
2	2.17% (n = 2)
1	0% (n = 0)
0	0% (n = 0)

**Table 3:** Multinomial regression results of the correlation between the level of knowledge (number of correct answers) and sociodemographic variables.

	Χ^2^=	*p*=
**Gender**	0.949	0.330
**Marital status**	0.174	0.677
**Source of Knowledge**	1.978	0.852
**Level of Education**	14.465	0.002 *

* = statistically significant.

## Data Availability

The data presented in this study are available on request from the corresponding author.

## References

[1] Benjafield A.V., Ayas N.T., Eastwood P.R., Heinzer R., Ip M.S.M., Morrell M.J., Nunez C.M., Patel S.R., Penzel T., Pépin J.L.D. (2019). Estimation of the Global Prevalence and Burden of Obstructive Sleep Apnoea: A Literature-Based Analysis. Lancet Respir. Med..

[2] Sateia M.J. (2014). International Classification of Sleep Disorders-Third Edition Highlights and Modifications. Chest.

[3] Eckert D.J., Malhotra A., Jordan A.S. (2009). Mechanisms of Apnea. Prog. Cardiovasc. Dis..

[4] Eckert D.J. (2018). Phenotypic Approaches to Obstructive Sleep Apnoea—New Pathways for Targeted Therapy. Sleep Med. Rev..

[5] Turnbull C.D., Stradling J.R. (2023). Endotyping, Phenotyping and Personalised Therapy in Obstructive Sleep Apnoea: Are We There Yet?. Thorax.

[6] Bosi M., Incerti Parenti S., Sanna A., Plazzi G., De Vito A., Alessandri-Bonetti G. (2021). Non-Continuous Positive Airway Pressure Treatment Options in Obstructive Sleep Apnoea: A Pathophysiological Perspective. Sleep Med. Rev..

[7] Incerti Parenti S., Bortolotti F., Alessandri-Bonetti G. (2019). Oral Appliances for Obstructive Sleep Apnea. J. World Fed. Orthod..

[8] Sowho M., Amatoury J., Kirkness J.P., Patil S.P. (2014). Sleep and respiratory physiology in adults. Clin. Chest Med..

[9] Mezzanotte W.S., Tangel D.J., White D.P. (1992). Mechanisms of Control of Alae Nasi Muscle Activity. J. Appl. Physiol..

[10] Fogel R.B., Trinder J., White D.P., Malhotra A., Raneri J., Schory K., Kleverlaan D., Pierce R.J. (2005). The Effect of Sleep Onset on Upper Airway Muscle Activity in Patients with Sleep Apnoea versus Controls. J. Physiol..

[11] Tangel D.J., Mezzanotte W.S., White D.P. (1991). Influence of Sleep on Tensor Palatini EMG and Upper Airway Resistance in Normal Men. J. Appl. Physiol..

[12] Wiegand L., Zwillich C.W., Wiegand D., White D.P. (1991). Changes in Upper Airway Muscle Activation and Ventilation during Phasic REM Sleep in Normal Men. J. Appl. Physiol..

[13] Edwards B.A., White D.P. (2011). Control of the Pharyngeal Musculature During Wakefulness and Sleep: Implications in Normal Controls and Sleep Apnea. Head Neck.

[14] Jordan A.S., McSharry D.G., Malhotra A. (2014). Adult Obstructive Sleep Apnoea. Lancet.

[15] Heinzer R., Marti-Soler H., Haba-Rubio J. (2016). Prevalence of Sleep Apnoea Syndrome in the Middle to Old Age General Population. Lancet Respir. Med..

[16] Lyons M.M., Bhatt N.Y., Pack A.I., Magalang U.J. (2020). Global Burden of Sleep-Disordered Breathing and Its Implications. Respirology.

[17] Kapur V.K., Auckley D.H., Chowdhuri S., Kuhlmann D.C., Mehra R., Ramar K., Harrod C.G. (2017). Clinical Practice Guideline for Diagnostic Testing for Adult Obstructive Sleep Apnea: An American Academy of Sleep Medicine Clinical Practice Guideline. J. Clin. Sleep Med..

[18] Giles T.L., Lasserson T.J., Smith B.J., White J., Wright J., Cates C.J. (2006). Continuous Positive Airways Pressure for Obstructive Sleep Apnoea in Adults. Cochrane Database Syst. Rev..

[19] Mogell K., Blumenstock N., Mason E., Rohatgi R., Shah S., Schwartz D. (2019). Definition of an Effective Oral Appliance for the Treatment of Obstructive Sleep Apnea and Snoring: An Update for 2019. J. Dent. Sleep Med..

[20] Armeni P., Borsoi L., Costa F., Donin G., Gupta A. (2019). Cost-of-Illness Study of Obstructive Sleep Apnea Syndrome (OSAS) in Italy.

[21] Rapelli G., Pietrabissa G., Angeli L., Manzoni G.M., Tovaglieri I., Perger E., Garbarino S., Fanari P., Lombardi C., Castelnuovo G. (2022). Study Protocol of a Randomized Controlled Trial of Motivational Interviewing-Based Intervention to Improve Adherence to Continuous Positive Airway Pressure in Patients with Obstructive Sleep Apnea Syndrome: The MotivAir Study. Front. Psychol..

[22] Borsoi L., Armeni P., Donin G., Costa F., Ferini-Strambi L. (2022). The Invisible Costs of Obstructive Sleep Apnea (OSA): Systematic Review and Cost-of-Illness Analysis. PLoS ONE.

[23] Simmons M., Sayre J., Schotland H.M., Jeffe D.B. (2021). Obstructive Sleep Apnea Knowledge Among Dentists and Physicians. J. Dent. Sleep Med..

[24] Dillow K., Essick G., Sanders A., Sheats R., Brame J. (2017). Patient Response to Sleep Apnea Screening in a Dental Practice. J. Public Health Dent..

[25] Saglam-Aydinatay B., Uysal S., Taner T. (2018). Facilitators and Barriers to Referral Compliance among Dental Patients with Increased Risk of Obstructive Sleep Apnea. Acta Odontol. Scand..

[26] Sia C.H., Hong Y., Tan L.W.L., van Dam R.M., Lee C.H., Tan A. (2017). Awareness and Knowledge of Obstructive Sleep Apnea among the General Population. Sleep Med..

[27] American Academy of Sleep Medicine (2014). The International Classification of Sleep Disorders.

[28] Şentürk H., Eryilmaz M.A., Vatansev H., Pekgör S. (2019). Evaluation of Knowledge Level Related to Obstructive Sleep Apnea Syndrome. Niger. J. Clin. Pract..

[29] Henzell M., Knight A., Antoun J.S., Farella M. (2013). Social Media Use by Orthodontic Patients. N. Z. Dent. J..

[30] Scarpelli S., Alfonsi V., Mangiaruga A., Musetti A., Quattropani M.C., Lenzo V., Freda M.F., Lemmo D., Vegni E., Borghi L. (2021). Pandemic Nightmares: Effects on Dream Activity of the COVID-19 Lockdown in Italy. J. Sleep Res..

[31] Paiva T., Reis C., Feliciano A., Canas-Simião H., Machado M.A., Gaspar T., Tomé G., Branquinho C., Silva M.R., Ramiro L. (2021). Sleep and Awakening Quality during COVID-19 Confinement: Complexity and Relevance for Health and Behavior. Int. J. Environ. Res. Public Health.

[32] Ruiz-Herrera N., Díaz-Román A., Guillén-Riquelme A., Quevedo-Blasco R. (2023). Sleep Patterns during the COVID-19 Lockdown in Spain. Int. J. Environ. Res. Public Health.

[33] Mehrtash M., Bakker J.P., Ayas N. (2019). Predictors of Continuous Positive Airway Pressure Adherence in Patients with Obstructive Sleep Apnea. Lung.

[34] Bortolotti F., Corazza G., Bartolucci M.L., Incerti Parenti S., Paganelli C., Alessandri-Bonetti G. (2022). Dropout and Adherence of Obstructive Sleep Apnoea Patients to Mandibular Advancement Device Therapy: A Systematic Review of Randomised Controlled Trials with Meta-Analysis and Meta-Regression. J. Oral Rehabil..

[35] Fox S. (2011). Health Topics. Pew Internet and American Life Project. https://www.pewresearch.org/internet/2011/02/01/health-topics-2/.

[36] Fox S., Rainie L. (2000). The Online Health Care Revolution: How the Web Helps Americans Take Better Care of Themselves: Pew Charitable Trusts: Washington, DC, USA. https://www.pewresearch.org/internet/2000/11/26/the-online-health-care-revolution/.

[37] Weedmark D. Why Is Audiovisual Media Considered a Powerful Tool and Means of Communication?. https://smallbusiness.chron.com/audiovisual-media-considered-powerful-tool-means-communication-33541.html.

[38] Osman W., Mohamed F., Elhassan M., Shoufan A. (2022). Is YouTube a Reliable Source of Health-Related Information? A Systematic Review. BMC Med. Educ..

[39] Incerti Parenti S., Gamberini S., Fiordelli A., Bortolotti F., Laffranchi L., Alessandri-Bonetti G. (2023). Online Information on Mandibular Advancement Device for the Treatment of Obstructive Sleep Apnea: A Content, Quality and Readability Analysis. J. Oral Rehabil..

[40] Incerti-Parenti S., Bartolucci M.L., Biondi E., Fiordelli A., Paganelli C., Alessandri-Bonetti G. (2023). Online Audio-Visual Information on the Treatment of OSA with Mandibular Advancement Devices: Analysis of Quality, Reliability and Contents. Appl. Sci..

[41] Lichtblau M., Bratton D., Giroud P., Weiler T., Bloch K.E., Brack T. (2017). Risk of Sleepiness-Related Accidents in Switzerland: Results of an Online Sleep Apnea Risk Questionnaire and Awareness Campaigns. Front. Med..

